# Evidence for long-term efficacy of a membrane filtration device in rural villages in Ghana

**DOI:** 10.1038/s41598-024-55977-8

**Published:** 2024-03-04

**Authors:** Joseph Marfo Boaheng, Jochen G. Raimann, Philip Narh, Seth Johnson, Linda L. Donald, Harrison Kwame Mati, Friedrich K. Port, Nathan W. Levin

**Affiliations:** 1 Easy Water for Everyone, New York, NY USA; 2 Katz School of Science and Health, New York, NY USA; 3 Ghana Health Service, Ada, Ghana; 4 Data Analytics, Holland College, Charlottetown, Canada

**Keywords:** Membrane filtration, Hemodialyzers, Polysulfone, Water sources, Fecal coliforms, *E. coli*, Gravity powered, Environmental sciences, Nephrology

## Abstract

Drinking water contaminated by pathogenic micro-organisms increases the risk of infectious gastrointestinal disease which could potentially lead to acute kidney injury and even death, particularly amongst the young and the elderly. Earlier studies have shown a substantial reduction in the incidence of diarrheal disease over a period of one year using a polysulfone membrane water gravity-powered water filtration device. The current report is a continuation of these studies to assess the long-term effects of the innovative method on diarrheal incidence rates over a 4-year follow-up period. This follow-up study monitored the trend of self-reported diarrheal events in all households in the previously studied villages for 5 months, in the last half of each study year, using the same questionnaire utilized in the earlier study. Three villages that had no device yet installed served as controls. We computed monthly diarrheal incidence rates for all study years (standardized to per 100 person-months) and compared these to the pre-device incidence rate in 2018 and in the control group, using the Wilcoxon rank sum exact test. The average diarrheal incidence rates of 1.5 p100pm in 2019, 2.19 p100pm in 2021, and 0.54p100pm in 2022 were significantly different from an earlier study that reported 17.8 p100pm rates before the devices were installed in 2018, (all p-values < 0.05). Concomitantly, self-reported diarrheal infections were substantially higher in the “control villages” not yet having the filtration device installed (80.9, 77.6, and 21.5 per 100 pm). The consistent and large reduction in diarrhea incidence documents the long-term efficacy of the use of the membrane filtration device. This simple water purification method using gravity flow improves public health in remote regions with limited resources.

## Introduction

As shown in the World Health Organization's annual reports, diarrheal disease remains the most common cause of death in children younger than 5 years^1^. This has been attributed to inadequate access to clean drinking water for many in regions with limited resources. It is estimated that around 780 million individuals lack access to improved drinking-water^1^ with the elderly and children being the most vulnerable to develop adverse outcomes. Consequently, the United Nation’s Sustainable Development Goal (SDG) 6—Target 3 focuses on the accessibility of clean drinking water and aims to solve this problem. A lack of access to clean quality drinking water is identified as a major risk factor for diarrhea, which in low- and middle-income countries, is the most common cause of acute kidney injury. In the light of limited resources and the need for the immediate diagnosis and treatment of diarrhea needed, the result is often death, especially in the young and the elderly^2^. Microbiological contamination of drinking water can lead to acute gastrointestinal diseases causing diarrhea, vomiting and other gastrointestinal symptoms resulting in dehydration and consequent acute kidney injury^3^. These dynamics may be avoided with access to safe drinking water through the use of suitable water purification techniques. Access to safe drinking water is therefore critical to decrease water-related morbidities and mortality in remote communities in Sub-Saharan Africa.

Hemodialysis is one type of renal treatment that removes waste products from the blood, eliminates excess fluid, and stabilizes electrolytes (potassium, sodium, chloride, phosphate, magnesium and bicarbonate)^4^. The hemodialyzer consists of hollow fibers (in this case the membrane is polysulfone in a plastic casing. The hemodialyzer has two sections; one for dialysate (a solution of pure water, electrolytes, and salts outside the hollow fiber wall and the other for blood inside the hollow fiber tubing, both divided by a semi-permeable membrane that has very tiny pores allowing toxic substances and water to pass through the membrane. Historically, hemodialyzers were frequently reused in the patient care setting, however in recent years this has decreased significantly, and they are generally discarded after one use. It has also been proven that repurposed hemodialyzers are successful in producing clean water from water contaminated with pathogenic organisms^5–8^.

Easy Water for Everyone (EWfE) is a 501(c)(3) non-profit, non-governmental organization (NGO) in the United States, and Ghana, with collaborative relationships in Senegal, and Uganda (It uses a polysulfone membrane filtration device to purify contaminated water to support villages lacking external power sources with a gravity driven approach.). With the pore size of the hemodialyzer membrane, being 0.003 microns, the filtration of bacterial, viral, parasite contamination is achieved. Further, with an hourly output of processed water between 250 and 500 L, the methodology is suitable for providing clean water for small to medium-sized communities with up to 1000 villagers. The use of this device is simple and is sustainable over protracted periods of time, resulting in an improvement of health outcomes including diarrheal diseases. Earlier studies by Raimann et al.^7^ recorded an incidence rate of diarrhea per person-month (ppm) of 0.177 (17.7 per 100 person-months) before, and 0.047 after introducing the polysulfone membrane filtration device utilizing repurposed hemodialyzers as described above, beginning in 2018, in 4 remote island communities in Ghana. This represents a 72% reduction in diarrheal infections.

The current study investigates the long-term efficacy of the use of the polysulfone membrane filtration device from 2019 to 2022. Analyzing the trends of diarrhea incidence rate over the 3-year period provides evidence to support the long-term efficacy of the use of the membrane filtration device against infectious diarrhea.

## Methods

This study focused on four sites of the EWfE Project in Ghana in which the membrane filtration device (NUF 500, NUFiltration Caesarea, Israel) was installed in 2018. Self-reported diarrhea incidence was recorded using a questionnaire that was collected by local data collectors trained and employed by the organization.

### Study design and data collection

Due to the relatively small sizes of the study villages and their limited capacity to track their self-reported cases of diarrhea, this follow-up study monitored the trend of self-reported diarrheal events in households for at least 5 months after device installation in each study year, using a paper-based questionnaire in the same villages used by Raimann et al.^7^. This present paper used a post-installation paper-questionnaire to collect information on diarrheal events at the household level after the introduction of the membrane filtration device. Prior to interviewing any household head, we obtained written informed consent from the participants. The households were subsequently interviewed by trained data collectors. The study obtained ethical approval from the Ghana Health Service Ethics Review Committee (GHS-ERC) to protect confidentiality, anonymity, and privacy. The study further adhered to all applicable guidelines and regulations.

### Statistical analysis

The computed diarrheal incidence rates per 100 person-months (p100pm) for the study villages from 2019–2022 were compared to the pre-device incidence rate in Raimann et al’s 2020 paper^7^. It should be noted that about 2% of the individuals lost to follow-up were excluded from the calculation of the incidence rate, ensuring that only participants actively followed throughout the study period contributed to the numerator and denominator. Monthly diarrheal incidence rates were plotted to determine their trends over these years. The Wilcoxon rank sum exact test was used to compare the incidence rate difference of successes. A bacterial culture was performed to find out the differences in bacterial counts in the source and filtered water samples over the years. This microbial assessment was done to enhance the robustness of our conclusions regarding the incidence rate observed within households. The research conducted thorough analyses of total coliforms, fecal coliforms and *E. coli* cultures in water samples collected from the source water and filtered water using the repurposed membrane (hemodialyzer) filtration device. The Ghana Water Research Institute, serving as a reputable and independent laboratory, collaborated in the study by performing regular bacterial counts (cfu/100 ml) on source and filtered water samples used in the villages. Data is presented with 95% confidence intervals. A repeated analysis for the “control villages” was made and compared the results to the study villages.

## Results

**Figure 1:**
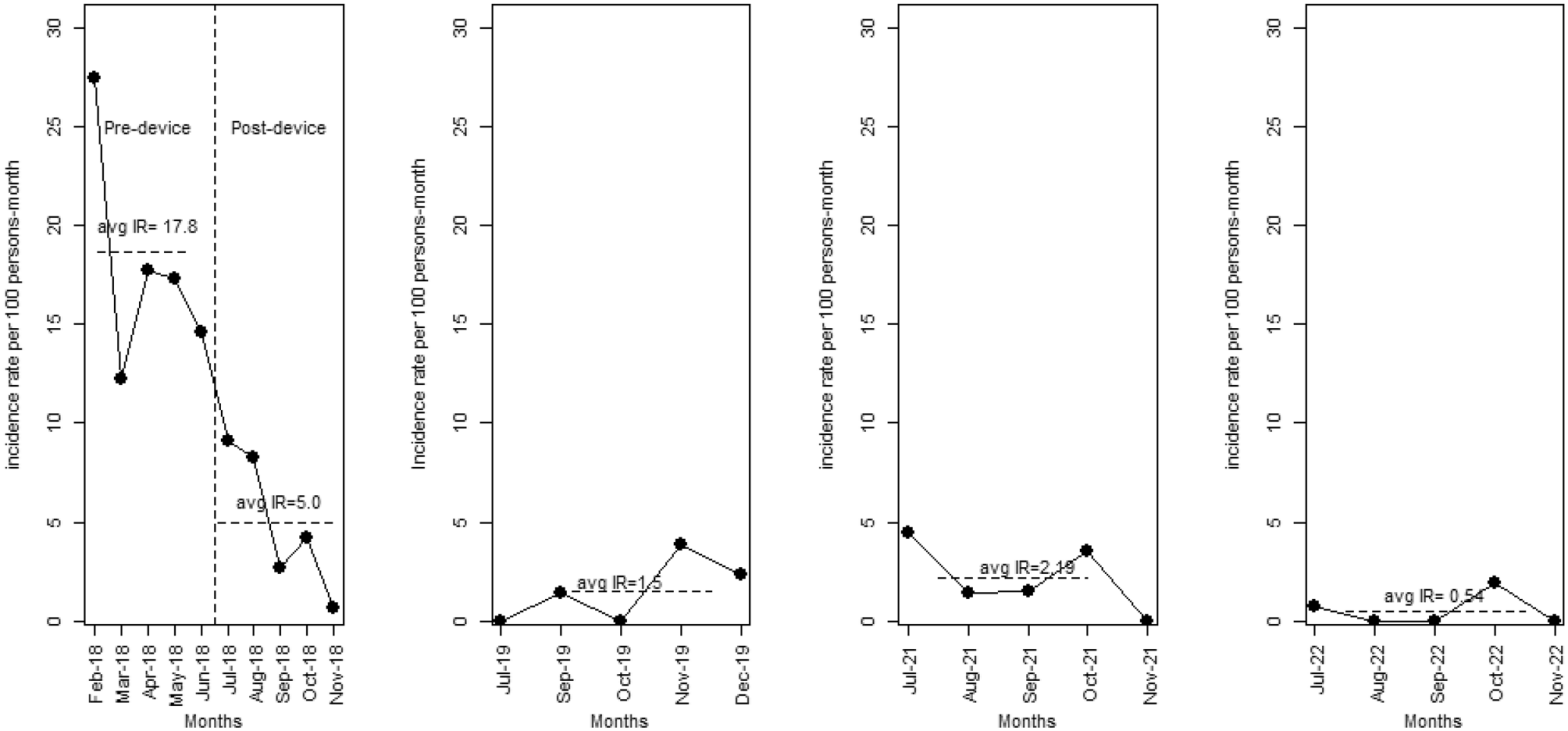
Trend of diarrhea cases over the 4-year period (2018 to 2022) in the study villages.

**Figure 2:**
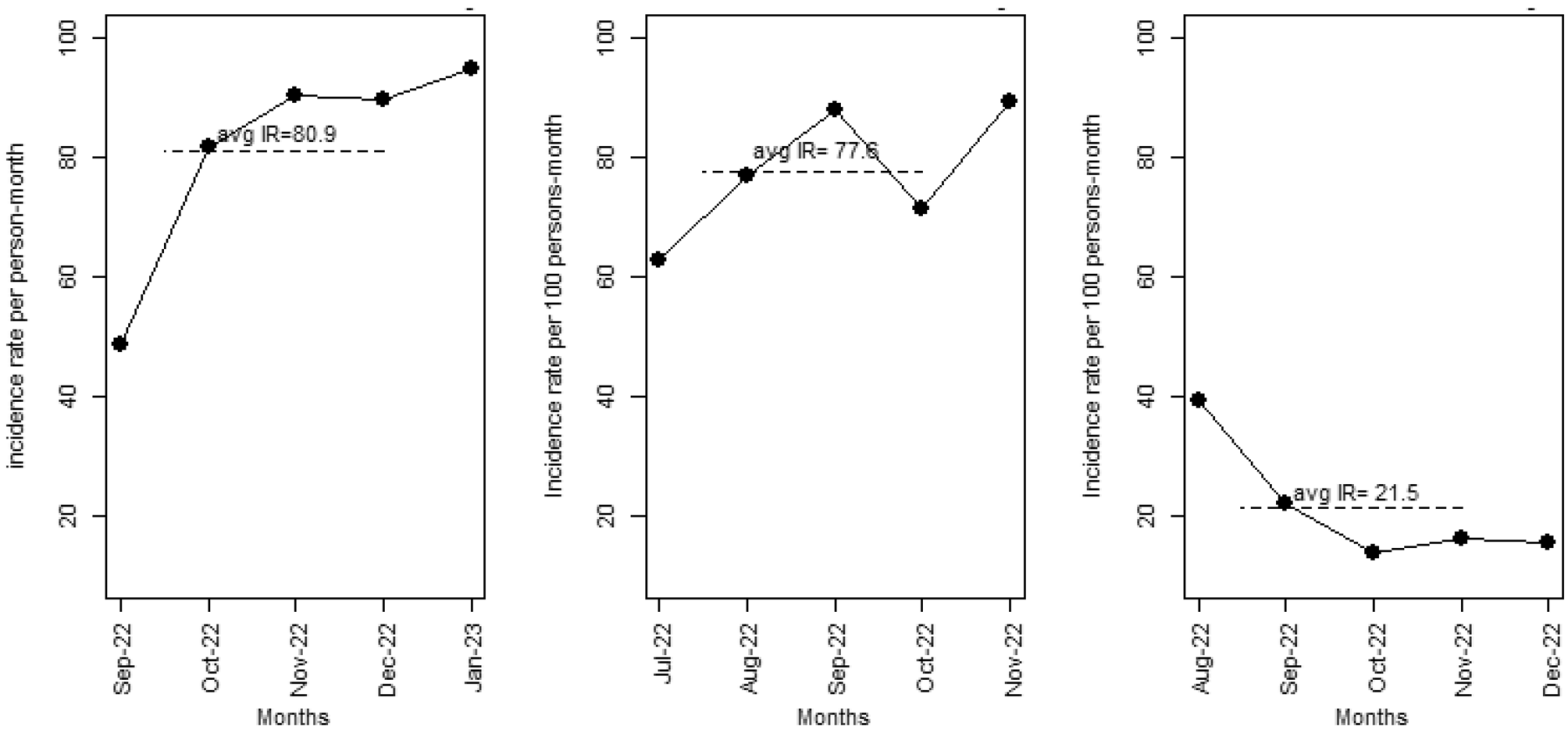
Trend of diarrhea cases in the control villages (Nkubeta, Tomefa, Asutifi).

The villages Agamakope, Alewusedekope, Anazome, and Baitlenya, located in the Ada-East district of Greater Accra region, Ghana, and its 441 inhabitants, (56% female, 96% practicing open defecation) were included in the analyses. The villages Nkubeta, Tomefa, and Asutifi, with a total of 1042 villagers (48% females and 40% practicing open defecation) served as “control villages”, i.e. villages without the polysulfone membrane filtration device. This is to confidently determine whether changes in diarrhea incidence rate or outcomes are due to the intervention, as opposed to being due to some other variable. In the study villages, the average diarrheal incidence rate for 2019 was 1.51 (1.64) per 100 person-months, while for 2021 and 2022 the rates were 2.19 and 0.54 per 100 person-months, respectively, all during post device installation phases (see Table 1 and Fig. 1). Our analysis produced statistically significant differences between the reference year (2018 mean incidence rate) and the mean incidence rate of 2019 (p-value = 0.012). The difference in mean incidence rates of 2018 and 2021 was statistically significant (p-value = 0.008) and comparison of similar tests of 2018 mean incidence rates vs that of 2022 also produced statistically significant differences (p-value = 0.011). The control sites reported high self-reported diarrhea incidence rates (see Fig. 2). The mean incidence rate for the Nkubeta site was 80.93 per 100 persons-month, Tomefa site recorded a mean incidence rate of 77.60 per 100 persons-month while Asutifi reported 21.48 per 100 persons-month.

**Table 1:** Diarrhea incidence rates before and by year after implementation of the membrane filtration device; test of mean difference.

Characteristic	2018 (before)	2019	2021	2022
Mean (SD)	17.86 (5.80)	1.51 (1.64)	2.19 (1.80)	0.5354 (0.85)
Median (IQR)	17.29 (14.56, 17.70)	1.39 (0.00, 2.32)	1.48 (1.45, 3.54)	0.00 (0.00, 0.74)
P-value vs before		0.012	0.008	0.011

The findings from the microbial assessment, detailing the median bacteria counts for each year, are summarized in Table 2. The counts are per 100 ml with IQRs shown in brackets. The results indicated that in 2019, the median total coliform counts before the utilization of membrane filtration device were 2398.5(732.0–5002.75) all of which became zero in the filtered water. Similarly, fecal coliform counts were 182.0(158.75–297.50) in the source water, with a complete absence in the filtered water. For *E. coli*, the counts were 92.5(65.25–170.5) in source water and became zero after the water was filtered with the membrane filtration device. The same trend as illustrated above was observed in 2020 and 2021.

**Table 2:** Microbial counts (with IQR) per 100 ml in source water and membrane filtered water.

	2019		2020		2021	
	Source	Filtered	Source	Filtered	Source	Filtered
Total coliforms	2399(732–5003)	0	82(25–146)	0	336(298–696)	0
Faecal coliforms	182(159–296)	0	11(9–20)	0	76(19–355)	0
*E. coli*	93(65–171)	0	4(2–8)	0	13(7–129)	0

## Discussion

The current study investigated the long-term efficacy of use of the hemodialyzer membrane filtration device (hemodialyzer) for water purification on the diarrheal infection rate in rural “last mile” communities in Ghana. The empirical results have provided evidence to support our earlier shorter-term findings^7^. The new empirical results for the long-term efficacy of the hemodialyzer membrane filtration device filtration device (hemodialyzer) showed that the rate of diarrheal infections at the household level was markedly and consistently reduced during the 4 years since the introduction of the hemodialyzer membrane filtration device filtration device in 2018. By contrast, the control villages showed very high rates of diarrheal infections in the same region over this period. This finding from the control villages strongly suggests that our hemodialyzer membrane filtration device filtration device made the difference in diarrheal reduction rather than any potential environmental factors in the region. The longer-term diarrheal incidence rates over 4 years with use of the filtered water to show a substantially greater reduction to an average diarrheal incidence rate of 2.19 or less which suggests a rate ratio of 0.12 i.e. a reduction of diarrhea by 88% than had been observed short term^7^. The microbial assessment carried out supports the results obtained at the household level, thereby strengthening the evidence for the efficacy of the hemodialyzers' filtration device in completely eradicating bacterial pathogens from drinking water.

The simplicity of the EWfE system with the repurposed hemodialyzer membrane filtration device hemodialyzer device has allowed a small team of village members to be trained to flush/backwash the hemodialyzer membranes thrice daily, in order to remove organic matter, and to pump the source water into the large, elevated tank. Thus, villages have become self-sufficient in managing clean water for long-term sustainability as reported^6^. Mothers having witnessed a large reduction in diarrhea, overall, particularly in their children, make sure that all maintenance is properly performed.

## Conclusion

The hemodialyzer membrane filtration device used by EWfE, utilizing repurposed hemodialyzers, has been shown to reduce the incidence of diarrhea over several years beyond the short-term results. These consistently large reductions in diarrheal incidence rates over the 4-year period after the implementation of the hemodialyzer membrane filtration device coupled with the results of bacterial culture assessment prove its long-term efficacy. Our study suggests that wider use of the device could improve public health in remote areas lacking external power sources.
